# Modeling Dendrite Coarsening and Remelting during Directional Solidification of Al-06wt.%Cu Alloy

**DOI:** 10.3390/ma17040912

**Published:** 2024-02-16

**Authors:** Ibrahim Sari, Nashmi Alrasheedi, Mahmoud Ahmadein, Joy Djuansjah, Lakhdar Hachani, Kader Zaidat, Menghuai Wu, Abdellah Kharicha

**Affiliations:** 1 Metallurgy Department, Montanuniversitaet of Leoben, Franz-Josef-Str. 18, 8700 Leoben, Austriamenghuai.wu@unileoben.ac.at (M.W.); 2 Department of Mechanical Engineering, Imam Mohammad Ibn Saud Islamic University, Riyadh 11564, Saudi Arabiajrdjuansjah@imamu.edu.sa (J.D.); 3 Department of Production Engineering and Mechanical Design, Tanta University, Tanta 31512, Egypt; m.ahmadein@f-eng.tanta.edu.eg; 4 Laboratoire Physique des Matériaux, Université Amar Telidji-Laghouat, Route de Ghardaia, BP 37G, Laghouat 03000, Algeria; 5 University Grenoble Alpes, Grenoble-INP, CNRS, SIMaP, 38400 Saint-Martin-d’Hères, France

**Keywords:** solidification, coarsening, volume average model, remelting, secondary dendrite arm spacing, coalescence

## Abstract

Research efforts have been dedicated to predicting microstructural evolution during solidification processes. The main secondary arm spacing controls the mushy zone’s permeability. The aim of the current work was to build a simple sub-grid model that describes the growth and coarsening of secondary side dendrite arms. The idea was to reduce the complexity of the curvature distribution with only two adjacent side arms in concurrence. The model was built and applied to the directional solidification of Al-06wt%Cu alloy in a Bridgman experiment. The model showed its effectiveness in predicting coarsening phenomena during the solidification of Al-06wt%Cu alloy. The results showed a rapid growth of both arms at an earlier stage of solidification, followed by the remelting of the smaller arm. In addition, the results are in good agreement with an available time-dependent expression which covers the growth and coarsening. Such model can be implemented as a sub-grid model in volume average models for the prediction of the evolution of the main secondary arms spacing during macroscopic solidification processes.

## 1. Introduction

It is well-known that crystal dendrites are formed during the solidification of metallic alloys by an advancing primary stalk accompanied by the formation of secondary arms, which undergoes a complicated ripening mechanism. Numerous researchers [1,2,3,4] have developed and validated predictive models for secondary dendrite arm spacing, incorporating various solidification parameters, including tip velocity and temperature gradient. The newly formed secondary arms grow competitively with respect to their neighbors (other side branches). Due to the curvature effect, some of them die (remelt) or stop growing. This mechanism is called the coarsening phenomenon and has been studied by many researchers [5,6,7,8]. In this paper, the term “coarsening” refers to the growth of solid regions of a low curvature in a liquid–solid mixture at the expense of regions of a higher curvature. Coarsening manifests itself in the solidification of metal alloys as the growth of larger dendrite arms with the simultaneous dissolution of smaller arms (ripening), filling the spaces in between dendrite arms (coalescence) and dendrites breakup (dendrite multiplication). The dendritic structures undergo a slow coarsening process when the surrounding melt reaches the equilibrium at a later stage. At this stage, the number of side-arms is reduced (retraction of small side branches towards their parent stem) and, consequently, the average microstructural length scale increases. I. Sari et al. [9] proposed a novel model that accurately predicts the secondary dendrite arm spacing (SDAS) based solely on the tip velocity and cooling rate during the directional solidification of different Pb-Sn alloys. The model involved a growing cylinder inside a liquid cylindrical envelope. The initial cylinder radius was assumed to be equal to the dendrite tip radius. However, the cylindrical envelope maintained a fixed radius in the order of the dendrite tip diffusion length. They found that for lower initial concentrations and slower cooling rates, coarser arms were developed. However, the predicted SDAS values decreased with increasing initial concentration. The validation was in excellent agreement with available measurements in terms of SDAS and tip velocity prediction. H. Neumann et al. [10] presented a radiographic analysis of the growth and coarsening of dendrites in a low-melting-point Ga-In alloy. Their measurements provide real-time in situ data on two phenomena that are of great importance in the coarsening of dendrites: sidearm retraction and pinch-off.

Q. Zhang et al. [11] used a 3-D CA model developed for the simulation of dendritic coarsening of alloys caused by the simultaneous melting and solidification phenomena. They observed that the dendritic microstructure gradually became coarser as the isothermal holding progressed. N. J. Whisler and T. Z. Kattamis [12] developed a model for dendritic coarsening during the solidification of Al-4.5wt%Cu alloy. The authors assumed the following: the deposition of solidified material on large arms, and the applicability of the Scheil equation to the solidification, dissolution, and shrinkage of small dendrite arms. The authors reported that solidification growth contributes more to the decrease in the surface-to-volume ratio Sv than does coarsening. In addition, coarsening contributes to the remelting of dendrite arms and enhances the solidification growth effect on Sv. Their model was in good agreement with an available experimental measurement in terms of the time evolution of Sv. In ref. [13], the authors proposed a numerical model to evaluate the microstructural and compositional data of binary alloys during and after solidification. This model incorporates the effects of solid-state diffusion and dendrite arm coarsening on solute distribution, leading to predictions that closely match experimental observations. Particularly, [13] demonstrates excellent agreement between measured and predicted cooling curves and local solidification times for binary aluminum alloys containing up to 10 wt% Mg and 6 wt% Zn. C M.G. Rodrigues et al. [14] simulated Bridging capillary-driven fragmentation and grain transport using a mixed columnar-equiaxed solidification model. However, the implementation of the interfacial area density was validated against the phase-field simulations of Neumann-Heyme et al. [15].

**Figure 1:**
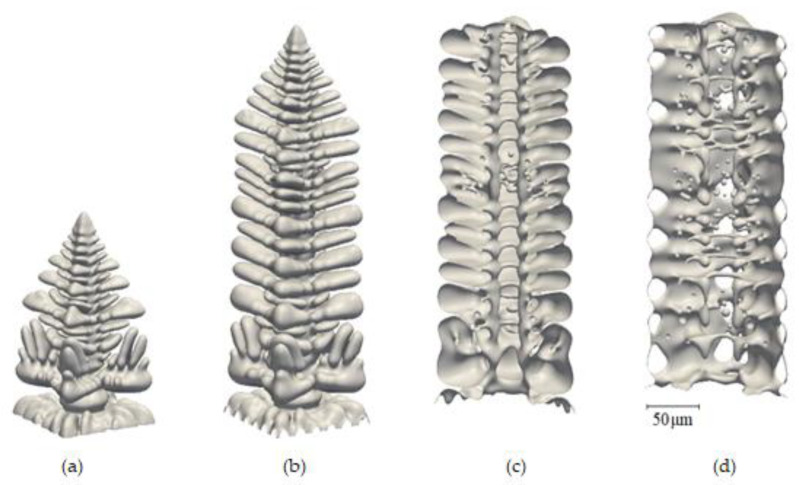
Evolution of the dendrite geometry for the base case simulation: full view of the growing dendrite at (**a**) 0.5 s and (**b**) 1 s; cutaway view of half of the dendrite at (**c**) 2.5 s and (**d**) 7 s from ref. [16], which is available under Creative Commons by Attribution 3.0 (CC BY 3.0) license.

Nevertheless, Neumann et al. [16] adopted a columnar dendritic solidification model using a 3D phase field simulation to analyze the concurrent growth and coarsening during the directional solidification of Al-06wt%Cu alloy. The model configuration was similar to the Bridgman experiment where dendrites grow in a fixed temperature gradient that moves at constant velocity. Indeed, they mentioned that there was an evolution of the dendrite shape during solidification as well as coarsening and coalescence of the side branches, as shown in Figure 1. They presented four different snapshots of the computed dendrite at different times. Initially, there is a rapid increase in the interfacial area by the formation of secondary and tertiary dendrite arms as shown in Figure 1a,b; then, coarsening and coalescence of side branches can be observed at t = 2.5 s (see Figure 1c), and at a high solid fraction, liquid channels are formed inside the solid structure (Figure 1c,d). They analyzed the coarsening phenomenon of the dendritic side branches during the solidification and, to some extent, coalescence (Figure 1b,c), beginning from the dendrite tip. Two major factors contribute to the coarsening and coalescence of secondary arms: increasing the solid fraction driven by mass transport from the liquid and the dissolution of the smaller arms. Furthermore, as the spaces between dendrite arms reduce, the coalescence becomes the dominant coarsening mechanism (i.e., branches’ radii change with time, and their counts decrease). On the other hand, M. C. Flemings [17] assumed that the “nucleant multiplication mechanism” can increase the number of nucleating particles and decrease it through coalescence. M. Chen and T. Z. Kattamis [18] studied dendrite coarsening during the directional solidification of Al-Cu–Mn alloys. They mentioned that the isothermal coarsening rate increases with temperature and decreases with time. They found that the temperature coarsening rate decreases with increasing copper concentration. Also, during solidification, the secondary dendrite arm spacing decreases with growth rate and increases with time.

To our opinion, the coarsening phenomenon may be one of the parameters that can influence the appearance of the freckles due to the coalescence of the side branches that can entrap liquid inside the solid. Therefore, the prediction of the local coarsening remains a major goal in the simulation of solidification processes. In the present study, a numerical model was developed to evaluate the growth/remelting and concentration evolution during the coarsening phenomenon of the solidification of Al-06wt%Cu alloy. The model aims to reduce the complexity of the curvature distribution with only two adjacent side arms in concurrence. Therefore, a system of equations is presented to address the coarsening phenomenon that includes the growth of the arms with time, the time evolution of the concentration (in the case of solidification and remelting), and the mixture concentration of the coarsening phenomenon.

## 2. Hypothesis

As mentioned previously, Neumann et al. [15,16] analyzed the coarsening phenomenon during the solidification of Al-06wt%Cu alloy. Neumann et al. [15,16] reported that the solid–liquid interface area, A, per volume of the enclosed solid phase, $V_{s}$, defines the specific interface area as SS=AVS. They mentioned that the evolution of the main inverse specific interface area under isothermal conditions can be defined by the following equation for surface energy-driven coarsening [15]:

(1) $$S_{s}^{-1}={\left(S_{s0}^{-n}+\mathrm{Kt}\right)}^{\frac{1}{n}}$$

where S_S0_, K, t, and n are the specific interface area at t = 0, coarsening rate constant, time, and coarsening exponent, respectively. The examination of the numerical and experimental data in [16] reveals that the coarsening exponent decreases with increasing cooling rate. However, the authors claim that an exponent of n = 3 is obtained for short times, while an exponent of n = 0.86 fits the simulation data for longer times. Also, the coarsening rate constant, K, is known to be a strong function of the solid fraction, which is discussed later.

Equation (1) presents the classical coarsening law. However, at a high cooling rate, when the solid volume fraction increases rapidly through the dendritic growth, Equation (1) is not suitable. For this reason, a time-dependent expression was proposed by Neumann-Heyme et al. [16], which involves the entire casting spectrum:

(2) $$S_{s}={\left(1-f_{s}\right)}^{r}{\left(S_{s0}^{-n}+\mathrm{Kt}\right)}^{-\frac{1}{n}}$$

where r is a fitting parameter that ranges from 0.2 to 1.35 in the current study. While in [15], experimental data from both solidification and isothermal coarsening indicate an exponent r of 0.25, phase-field simulations, assuming a highly regular and symmetric dendrite arrangement, require a higher r of 0.4 for a better fit. f_s_ is the solid fraction which is defined as the volume sum of the arms over the total volume ($f_{S}=\frac{\left(V_{1}+V_{2}\;\right)}{V_{\mathrm{total}}}$).

Diffusional interactions and interface coalescence, which become more significant as the solid volume fraction gets closer to unity, are taken into consideration in the first term on the right-hand-side, and the strength of both processes is distinguished by the exponent r.

**Figure 2:**
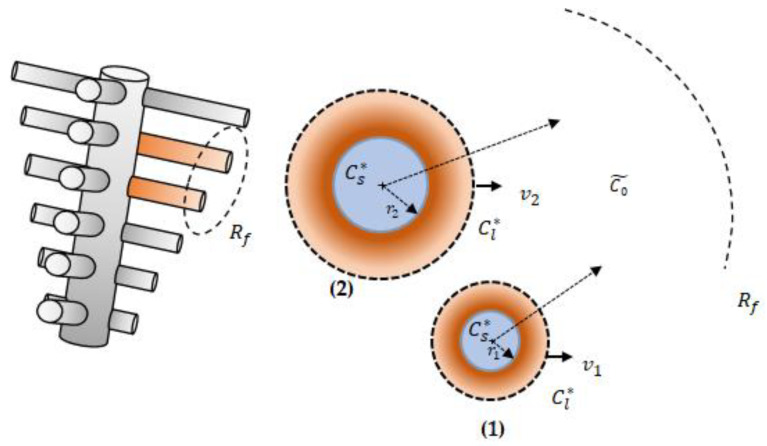
Schematic representation of the dendrite side branches which are simplified as cylinders (**left**). Side view of two competitively growing secondary dendrite arms during the solidification of Al-06wt%Cu alloy (**right**), where (1) and (2) refer to the small and the big arms, respectively.

**Figure A1:**
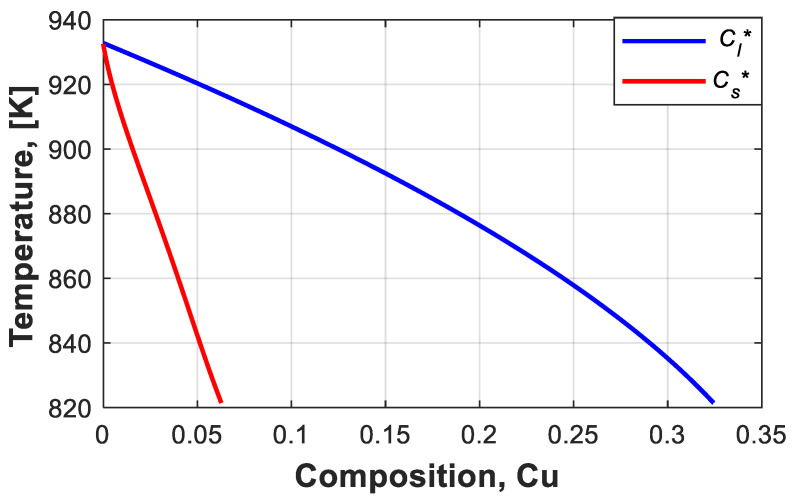
Phase diagram of Al-Cu alloy; liquidus and solidus curves are presented in blue and red.

As the morphology of the columnar dendrites is complex, the side branches are simplified with a cylindrical interface as shown in Figure 2 in a similar way to the assumptions of Wu et al. [19]. Two side branches are presented in Figure 2: one with a small initial radius $r_{1}$ and a larger one with an initial radius $r_{2}$. The arms are assumed to grow perpendicularly to the dendrite trunk inside an enveloped cylindrical volume with radius $R_{f}$. At the liquid/solid interface, thermodynamic equilibrium concentrations, $C_{l}^{*}$, $C_{s}^{*}$, are assumed, where $C_{l}^{*}$, $C_{s}^{*}$ represent the liquidus and solidus concentrations, respectively, are defined in Equations (A1), (A2) and Table A1 and plotted in Figure A1 (more details are in Appendix A). The liquid average concentration ($\tilde{C_{0}}$) covers the liquid region inside the enveloped volume. Initially, the value of the average liquid concentration is 06wt% of Copper, and it increases with time during the solidification as the solute is rejected in the liquid region. At t =0 s, the arms have the same initial temperature, but they have different initial radii (one is small and the other is large), and the radii change with time (grow and/or remelt). The thermophysical properties of Al-06wt%Cu alloy are provided in Table 1.

## 3. The Growth Model

The growth rate of the cylinders in the current study is expressed by the time evolution of their radii $r_{1}$ and $r_{2}$, where $\frac{\partial r_{1}}{\partial t}=v_{1}$ and $\frac{\partial r_{2}}{\partial t}=v_{2}$ (as given by Equations (3) and (4)) are the growth rate of the arms 1 and 2, respectively. The liquid volume per unit length of dendrite arm ($V_{\mathrm{liq}}$) is defined as the enveloped cylinder volume minus the solid volume of the arms as expressed in Equation (5). In addition, the liquid average concentration ($\tilde{C_{0}}$) is defined in Equation (6), considering the assumption that equilibrium is always valid at the solid/liquid interface. However, the term ${c_{s}^{*}}_{\mathrm{melt}/\mathrm{soli}}$ presented on the right-hand-side of Equation (6) is the equilibrium solid concentration. This latter change depends on the growth rate, and it varies as follows:

- Positive growth rate in case of solidification (growth): ${c_{s}^{*}}_{\mathrm{melt}/\mathrm{soli}}=c_{s}^{*}$.
- Negative growth rate in case of remelting: ${c_{s}^{*}}_{\mathrm{melt}/\mathrm{soli}}=\tilde{C_{s}}$ see Equations (7) and (8)).

where $V_{1}={\pi r}_{1}^{2}\;$ and $V_{2}={\pi r}_{2}^{2}\;$ are the solid volumes per unit length of arms 1 and 2, respectively. Also, $S_{1}=2{\pi r}_{\;1}$ and $S_{2}=2{\pi r}_{2}$ are the surface areas per unit length of cylinders 1 and 2, respectively, and $V_{\mathrm{total}}={\pi R}_{f}^{2}$ is the total volume of the envelope per unit length. Furthermore, the mixture concentration of the coarsening phenomenon is defined as per Equation (9). Equations (3)–(9) were solved in MATLAB R2022b Software simultaneously through an iterative calculation to obtain the growth of the arms and their corresponding average solid concentration during the coarsening phenomenon. Our model predicts results at each time step. We assumed a variable time step that was adjusted to ensure that the arm’s radius had a change of less than 5% between consecutive time steps. The largest time step assumed in the simulation was 10^−3^ s (i.e., the maximum uncertainty was 10^−3^ s). This ensured numerical stability and accuracy while efficiently capturing the dynamic behavior of the arm’s growth process. The physical properties of Al-06wt%Cu alloy are presented in Table 1.

(3) $$\frac{\partial r_{1}}{\partial t}=\frac{D_{l}}{r_{1}}\;\frac{c_{l}^{*}-\tilde{C_{0}}-\frac{\Gamma }{m_{l}{\;r}_{1}}}{c_{l}^{*}-c_{s}^{*}}$$

(4) $$\frac{\partial r_{2}}{\partial t}=\frac{D_{l}}{r_{2}}\;\frac{c_{l}^{*}-\tilde{C_{0}}-\frac{\Gamma }{m_{l}{\;r}_{2}}}{c_{l}^{*}-c_{s}^{*}}$$

(5) $$V_{\mathrm{liq}}=\pi \left({R_{f}}^{2}-{r_{1}}^{2}-{r_{2}}^{2}\right)$$

(6) $$\frac{\partial \left(V_{\mathrm{liq}}\;\tilde{C_{0}}\right)}{\partial t}=-2{\pi r}_{1}.v_{1}\left({c_{s}^{*}}_{\mathrm{melt}/\mathrm{soli}}\;\right)-2{\pi r}_{2}.v_{2}\left({c_{s}^{*}}_{\mathrm{melt}/\mathrm{soli}}\right)$$

(7) $$\frac{\partial \left(V_{1}\;{\tilde{C_{s}}}_{1}\right)}{\partial t}=v_{1}\left({c_{s}^{*}}_{\mathrm{melt}/\mathrm{soli}}\right){\;S}_{1}$$

(8) $$\frac{\partial \left(V_{2}\;{\tilde{C_{s}}}_{2}\right)}{\partial t}=v_{2}\left({c_{s}^{*}}_{\mathrm{melt}/\mathrm{soli}}\right){\;S}_{2}$$

(9) $$C_{\mathrm{mix}}=\frac{V_{1}\;{\tilde{C_{s}}}_{1}+V_{2}\;{\tilde{C_{s}}}_{2}+V_{\mathrm{liq}}\tilde{C_{0}}}{V_{\mathrm{total}}}$$

## 4. Results and Discussion

**Figure 3:**
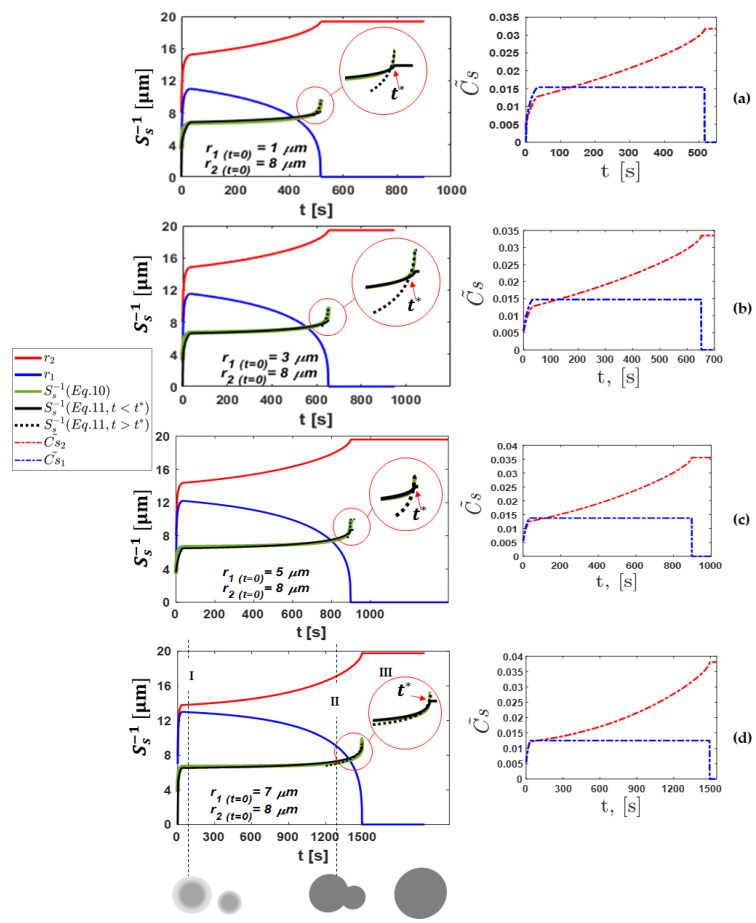
Left: inverse specific interface area and arm’s radius vs. time. Right: the average solid concentration vs. time of both arms for various initial values of the small arm (r_1_ = 1,3,5, and 7 μm) are presented in (**a**–**d**). The solid grey circles at the lower-left side are the schematic of the large and small dendrite arms. They initially grow concurrently and coalesce; finally, the larger arm grows and the smaller one remelts.

Based on the iterative equations system given in Equations (3)–(9), the growth and evolution of the concentration of the big and small arms during the coarsening/remelting of Al-06wt%Cu alloy were calculated. The results of the numerical solution are presented in Figure 3. Initially, at t=0 s, the arms had the same temperature conditions. The time evolution of the arms during the growth and/or remelting for different initial arm radii of the small arm ($r_{1}=1,\;3,\;5,\;\mathrm{and}\;7\;\mu m$) are presented in Figure 3a, b, c, and d, respectively. The initial radius of the big arm was set to $\;r_{2}=8\;\mu m$. The corresponding $\tilde{C_{s}}$-time curves are also shown in the right column of Figure 3a–d. It is obvious that the radii of both the small and big arms vary in accordance with Equations (3) and (4), respectively. Furthermore, the solid average concentrations of both arms change with respect to Equations (7) and (8). In order to check the validity of the present iterative calculation, it is useful to connect Equation (10) with Equation (11) proposed by Neumann et al. [15]. Indeed, Equation (10) was proposed to calculate the inverse specific interface area, which is defined as the inverse of the solid–liquid interface area per volume of the arms, as follows:

(10) Ss−1=12πr1+2πr2πr12+πr22

The evolution of Equation (10) is represented in Figure 3 by green lines. Equation (11) represents the inverse specific interface area expression (inverse of Equation (2)) multiplied by a corrective factor (A) which was used to fit the numerical results, [15] and it is defined as follows:

(11) $$S_{s}^{-1}=A\;{\left(1-f_{s}\right)}^{-r}{\left(S_{s0}^{-n}+\mathrm{Kt}\right)}^{\frac{1}{n}}$$

where A is a corrective factor (see Table 2).

Three different stages are presented in Figure 3 and noted by I, II, and III, which present different steps of the process. In stage I: both arms are growing. In Region II, the arms coalesce with the remelting of the small arm. In Region III, the small arm disappears, and only one arm survives. According to the results presented in Figure 3, one can easily notice that the small arm grows at an earlier stage of the coarsening phenomenon, and then it remelts (dies) until it disappears because of the curvature undercooling effect ($∆T_{r}=\Gamma /(m_{l}r_{1})$). On the other hand, the radius of the large arm gradually increases until it asymptotically reaches a value lower than the final radius of the enveloped volume,${\;R}_{f}.$ In the current analysis, $R_{f}$ was set to 20 μm, which represents about one-fifth the primary dendrite arm spacing (${\;R}_{f}\approx 1/5{\;\lambda }_{1}$).

The results of our model are presented in Figure 3a–d, which show the time evolution of the radius and concentration of the small and big arms represented by blue and red profiles, respectively. The initial selected arm’s radius at t = 0 s is a very important parameter that directly influences the coarsening time. As evident in the left column of Figure 3a–d, increasing the initial small arm’s radius prolongs the coarsening stage. The coarsening stage depends on how long the small arm remains. A small coarsening stage (about 516 s of the surviving time of the small arm) is obtained, as shown in Figure 3a, with a selected initial small arm’s radius of ${\;r}_{1\;\left(t=0\right)}=1\;\mu m$. However, the coarsening stage increases to 652 s, 900 s, and 1492.6 s as the initial small arm’s radius increases to 3, 5, and 7 μm, respectively. This can be simply justified as follows: the larger the small arm’s radius, the longer the time of the concurrent growth between the arms and the re-melting of the small arm (i.e., the surviving time of the small arm becomes longer). In addition, the small arm’s radius (blue curve) grows faster than the big arm’s radius at the first stage of the growth. For example, in Figure 3a, a growth of $∆r_{1}=9.99\;\mu m$ is achieved within the first 33 s. However, a slightly slower growth is noticed for the big arm $∆r_{2}=7.21\;\mu m$ within the same period.

It is noteworthy that as the arm grows (solidification case), the solid concentration at the liquid–solid interface changes according to the equilibrium phase diagram. However, when the re-melting occurs, the solid concentration follows its earlier path (instantaneous earlier concentration). Therefore, an average solid concentration is assumed inside the solid, which is $\left(\tilde{C_{s}}\right)$ as defined in Equations (7) and (8) and presented in the right column of Figure 3a–d. Then, in the re-melting case, the solid concentration changes by what was already solidified before. In the right column of Figure 3a–d, at stage I, the solid average concentration of the small arm (blue dashed line) is higher than that of the big arm (red dashed line). This justifies the rapid growth of the small arm than the big arm, as discussed above.

A zoomed-in view marked by red circles on the insets of the left column of Figure 3a–d highlights the evolutions of the inverse specific interface area during the final stage of the coarsening phenomenon, specifically a few moments before the disappearance of the small arm. At this stage, a sharp increase in the inverse specific interface area (green profile) occurs due to the rapid re-melting of the small arm and the fast growth of the big arm. Consequently, the transition from stage II to stage III occurs in the interface area, as depicted in the sketch below Figure 3. This transition is characterized by a shift from a two-attached-circle interface configuration to a single-circle interface configuration. This behavior can be called a critical phase, i.e., the inverse specific interface area changes from a complex shape to a circular shape. For this reason, it is difficult to adjust the ideal parameters of the proposed time-dependent expression given by Neumann et al. [15] to fit the present results (green profile). For this reason, the inverse interface area was separated into two stages by a critical time, “t*”, (see the inset of Figure 3a–d). Indeed, the critical time depends on the re-melting time of the small arm, and it is about 493 s, 624 s, 887 s, and 1488 s in Figure 3a, b, c, and d, respectively. In this study, a constant cooling rate (CR) of 3 K/s was applied during the solidification of the Al-6wt.pct Cu alloy, using the parameters outlined in Table 2. The proposed time-dependent expression given by Neumann et al. [15] (Equation (11)), which is presented by the black profiles (solid and dashed curves), provides a good fitting to the present results presented by the green profiles, as shown in Figure 3a–d. However, with $K=1\times 10^{-23}{\;\mu m}^{3}{\;s}^{-1}$, r = 0.3 and n = 3, Equation (11) (solid black line) provides a good fit to Equation (10) only in stages I and II at the growth stage and coarsening of the side-branches (t < t*), except in Figure 3d. However, by using the same parameter, the solid black profile does not fit the present results (green curve) when t > t*. This suggests that the proposed expression is not yet fully capable of capturing the complex behavior of the inverse-specific interface area during the complete coarsening process. In contrast, for $K=1\;\times 10^{-23}{\;\mu m}^{3}{\;s}^{-1}$, r = 0.3 and n = 0.83, Equation (11) (dashed black line) exhibits a better fit to Equation (10) when the time exceeds the critical time t* in all the analyzed cases, indicating improved accuracy during the final stage of coarsening. Indeed, H. Neumann-Heyme et al. [14] reported that r = 0.4 was used for the simulations and r = 0.25 for the experiments. The coarsening exponent (n) and fitting parameter (r) differed significantly in the case with an initial arm radius of r = 7 μm, compared to other cases. This difference arises from the intense competition in growth between the arms due to their nearly identical initial conditions (only 1 μm difference with the bigger arm). This competition was significantly stronger and more prolonged compared to the other cases, where the smaller arm remelted faster. Additionally, the transition from two coalescing arms to a single circular interface (shown schematically in Figure 3) varies across cases, making it challenging to fit the data using constant values of n and r for all the studied cases. Ref. [14] reports a large uncertainty in the exponent (r) due to limited measurement ranges and focuses solely on solid fractions under 0.4. This suggests that (r) is mainly influenced by dendrite growth rather than coarsening. The simulation results presented in ref. [14] show a higher (r) value (0.4) compared to the experiments (r = 0.25), which is likely due to the highly ordered and symmetric arrangement of columnar dendrites. This arrangement promotes faster interface coalescence, changing the interfacial area and leading to a higher (r). In contrast, equiaxed dendrites in the experiment grow freely without a temperature gradient, resulting in random orientations. Notably, the fitting parameter r = 0.2 in case (d), which is predicted by our model (Figure 3) and used for longer time, is very close to the value obtained experimentally [14]. The precise influence of dendrite orientation and spacing on (r) requires further investigation. In addition, a coarsening rate constant of K = 23.5 ${\mu m}^{3}{\;s}^{-1}$ was used by H. Neumann-Heyme et al. [14] and C. M.G. Rodrigues et al. [12]. Depending on the alloy, a wide range of the coarsening rate constant, which is an alloy-dependent fitting constant, has been used in the literature. Zhiyong Cai et al. [7] presented a different coarsening rate (K) constant for the Al-Si alloys at various annealing temperatures, which was in the range of $10^{-14}$ ${\mu m}^{3}{\;s}^{-1}$. The authors [7] identified several factors influencing parameter K, including particle size and cooling rate. Notably, they reported that the coarsening rate K decreases with decreasing cooling rate. In their study, K falls within the range of 10^−14^ for a cooling rate (CR) of 6 × 10^5^ K/s. In our model, the predicted K was found to be in the range of 10^−19^ to 10^−23^, which corresponds to our lower cooling rate of CR = 3 K/s, which aligns reasonably with this trend. Also, A. Baldan [20] presented the coarsening rate constant, K, for different Sn-Pb, Pb-Sn, Fe-Cu, and Co-Cu alloys; however, the range was from 0.5 to $20\times 10^{-17}$ $m^{3}{\;s}^{-1}$.

Figure 3d clearly illustrates that stage I is characterized by a sharp increase in both arms’ radii. This rapid growth, in turn, results in a corresponding rise in the specific interface area, which is, in fact, the periphery of the arms. This is caused by the initial free dendritic growth and side branches, creating a large interfacial area while the melt is still undercooled. However, at stage II, due to the coalescence of the arms (as presented in the middle sketch below Figure 3), the specific interface area curve (green curve) is almost a horizontal line ranging approximately from 6.5 to 7.2 μm. This stability is attributed to a nearly balanced exchange between the growth and re-melting of the arms. The corrective factor A remains close to unity during stages I and II but exhibits a significant decrease at the onset of stage III. It is interesting to note that the present value of ${S_{s0}}^{-1}$ = 3.3 μm is close to the steady-state primary dendrite tip radius of 2.7 μm as reported by Neumann et al. [15].

Later, in stage III, the coarsening process does not exist, and only one arm survives. The comparison between the present numerical results and the proposed expression of the phase field model [15] shows that they are in good agreement. Indeed, the fitting parameters adopted in the proposed time-dependent expression derived by Neumann et al. [15] demonstrate good agreement with the inverse specific interface area when the time is less than the critical time (t < t*). However, using the same parameters, Equation (11) fails to maintain this satisfactory agreement with the inverse specific interface area when the time is beyond the critical time (t > t*). This highlights the importance of selecting appropriate values for the fitting parameters “r” and “n” to achieve an accurate modeling of the inverse-specific interface area across the entire coarsening process.

## 5. Conclusions

The present study addressed the coarsening phenomenon during the solidification of the Al-06wt%Cu alloy. The relatively simple numerical model showed its effectiveness in predicting the coarsening phenomenon in terms of the growth/melt of the side branches and the evolution of the concentration during the solidification and melting processes. Also, the inverse specific area parameters predicted by our model are in excellent agreement with those predicted with the empirical filling relation extracted from phase-field simulations. This paper shows that the two arms in competition seem to be enough to capture most of the evolution of coarsening. This model can be implemented in a volume-average model for the prediction of the microstructural evolution as a function of the tip radius. The model can also be implemented inside a macroscopic volume-average model to explore the effects of coarsening on the mushy zone’s permeability for various solidification models.

## Figures and Tables

**Table 1:** Thermophysical properties and initial conditions used in the calculation of coarsening/remelting of Al-06wt%Cu alloy [16].

Properties	Description	Value
$C_{0}$	Initial concentration of alloy.	0.06
CR	Cooling rate, (K/s).	3
$T_{l}$	Liquidus temperature, (K).	918.5
$T_{s}$	Solidus temperature, (K).	821.4
∆T	Undercooling, (K).	0.5
Γ	Gibbs Thomson coefficient, (m K).	$2.4\times 10^{-7}$
$D_{l}$	Liquid diffusion coefficient, (m^2^ s^−1^).	$3\times 10^{-9}$
$R_{f}$	Final radius of the enveloped volume, (µm).	20

**Table 2:** The parameters used in the fitting of $S_{s}^{-1}$ (Equation (11)) for the solid and dashed lines as presented on the second column and as plotted in Figure 3a–d.

Figure 3		r	n	$S_{s0}^{-1}\;\left[\mu m\right]$	$K\;\left[\frac{{\mu m}^{3}}{s}\right]$	A	$r_{1\;\left(t=0\right)}\left[\mu m\right]$	$r_{2\;\left(t=0\right)}\left[\mu m\right]$
a	**––––––**	0.3	3	3.3	$1\times \;10^{-23}$	1	1	8
	**---------**	1.35	0.83			0.066		
b	**––––––**	0.3	3			1.045	3	
	**---------**	1.35	0.83			0.06		
c	**––––––**	0.3	3			0.98	5	
	**---------**	1.35	0.83			0.04		
d	**––––––**	0.2	0.7		$1\times \;10^{-19}$	1.1	7	
	**---------**	0.3	0.83		$1\times \;10^{-23}$	0.805		

## Data Availability

Data are contained within the article.

## Nomenclature

$C_{0}$	Initial concentration of alloy
${\tilde{C_{s}}}_{1},$ ${\tilde{C_{s}}}_{2}$	Solid concentration of the small and big arms
$\tilde{C_{0}}$	Liquid average concentration
$c_{l}^{*}$ **,** $c_{s}^{*}$	Liquid and solid concentrations at the equilibrium
${c_{s}^{*}}_{\mathrm{melt}/\mathrm{soli}}$	Solid concentration at the equilibrium in melting and solidification cases
$\tilde{C_{s}}$	Solid concentration in melting case
$C_{\mathrm{mix}\left(\mathrm{coarsening}\right)}$	Mixture concentration
$T_{l}$	Liquidus temperature, (K)
$T_{s}$	Solidus temperature, (K)
∆T	Undercooling, (°C)
$m_{l}$	Liquidus slope, (K wt%^−1^)
$f_{s}$	Volume fraction of solid
CR	Cooling rate, (K/s)
$\lambda_{2}$	Secondary arm dendrite spacing, (µm)
Γ	Gibbs Thomson coefficient, (m K)
$D_{l}$	Liquid diffusion coefficient, (m^2^ s^−1^)
$r_{1}$, $r_{2}$	Initial radius of the small and big arms, (µm)
$R_{f}$	Final radius of the enveloped volume, (µm)
$v_{1}$, $v_{2}$	The growth velocities of the small and big arms, (m s^−1^)
$V_{\mathrm{total}}$	Volume of the enveloped cylinder per unit length, (m^2^)
$V_{1}$, $V_{2}$	Solid volume of the small and the big arms, (m^3^)
$V_{\mathrm{liq}}$	Volume of the liquid per unit length, (m^2^)
$S_{1},\;S_{2}$	Surface areas per unit length of the small and big arms, (m)

## Appendix A

(A1)
$$C_{l}^{*}\left(T\right)=p_{1}T^{4}+p_{2}T^{3}+p_{3}T^{2}+p_{4}T+p_{5}$$

(A2)
$$C_{s}^{*}\left(T\right)=q_{1}T^{4}+q_{2}T^{3}+q_{3}T^{2}+q_{4}T+q_{5}$$

**Table A1:** The corresponding constants of the liquidus and solidus concentration of the Al-Cu phase diagram.

$C_{l}^{*}$ (T)				
$p_{1}$	$p_{2}$	$p_{3}$	$p_{4}$	$p_{5}$
1.8778.10^−11^	−3.89535.10^−8^	1.7934.10^−5^	0.0014739	−1.152
		$C_{s}^{*}$ (T)		
$q_{1}$	$q_{2}$	$q_{3}$	$q_{4}$	$q_{5}$
3.8154.10^−10^	−9.065.10^−7^	8.0682.10^−4^	−0.31942	47.585
